# Red Blood Cell Count: An Unrecognized Risk Factor for Nonalcoholic Fatty Liver Disease

**DOI:** 10.3389/fendo.2021.760981

**Published:** 2021-12-07

**Authors:** Fang Zhong, Liying Guan, Haiyan Lin, Meng Zhao, Yiming Qin, Qihang Li, Zhongshang Yuan, Guang Yang, Ling Gao, Jiajun Zhao

**Affiliations:** ^1^ Department of Endocrinology, Shandong Provincial Hospital, Cheeloo College of Medicine, Shandong University, Jinan, China; ^2^ Shandong Clinical Medical Center of Endocrinology and Metabolism, Jinan, China; ^3^ Shandong Institute of Endocrine and Metabolic Disease, Jinan, China; ^4^ Health Management Center, Shandong Provincial Hospital Affiliated to Shandong First Medical University, Jinan, China; ^5^ Department of Endocrinology, Shandong Provincial Hospital Affiliated to Shandong First Medical University, Jinan, China; ^6^ College of Chemical Engineering and Materials Science, Shandong Normal University, Jinan, China; ^7^ Department of Biostatistics, School of Public Health, Shandong University, Jinan, China; ^8^ Beijing Institute of Pharmacology and Toxicology, Beijing, China

**Keywords:** nonalcoholic fatty liver disease, red blood cell, indicator, risk factor, longitudinal cohort study, generalized estimating equation

## Abstract

**Objective:**

Nonalcoholic fatty liver disease (NAFLD) is becoming a global public health challenge. A convenient NAFLD indicator will greatly facilitate risk appraisal and prevention. As a readily available and inexpensive hematological index in routine clinical examinations, red blood cells (RBCs) are gaining increasing attention in many diseases, such as metabolic syndrome, but their association with NAFLD is unknown.

**Methods:**

This health management cohort study included 27,112 subjects (17,383 non-NAFLD and 9,729 NAFLD) with up to 5 years of follow-up (median 2.8 years). NAFLD was diagnosed by ultrasonography. NAFLD severity was categorized as mild, moderate, or severe. The generalized estimation equation (GEE), an extension of generalized linear models that allows for analysis of repeated measurements, was used to analyze the association between RBC count and NAFLD.

**Results:**

Overall, 4,332 of 17,383 (24.9%) subjects without NAFLD at baseline developed NAFLD. Incident NAFLD risk was positively associated with RBC count. After adjustment for hemoglobin and other confounders, the risk of incident NAFLD was 21%, 32%, and 51% higher in the second, third, and fourth RBC count quartiles, respectively, than in the lowest quartile. In 1,798 of 9,476 (19.0%) subjects with NAFLD at baseline, the severity of NAFLD increased. NAFLD progression risk increased progressively as RBC count increased (P for trend < 0.001). Every one-unit (10^12^ cells/L) increase in RBC count was associated with a 53% [OR 1.53 (95% CI 1.32-1.77)] increased risk for NAFLD progression.

**Conclusions:**

Elevated RBC count was independently associated with a high risk of NAFLD incidence and progression. This finding revealed a convenient NAFLD risk indicator.

## Introduction

Nonalcoholic fatty liver disease (NAFLD) encompasses a spectrum of diseases characterized by excessive fat accumulation in hepatocytes (1, 2). Due to dramatic lifestyle and dietary structure modifications in the past century, NAFLD prevalence has increased over time to a current rate of 40% (3). In addition to liver-related morbidity and mortality, NAFLD increases the risk of atherosclerotic cardiovascular disease, a variety of malignant tumors, and metabolic disorders such as type 2 diabetes, adding to the burden of disease (4–7). Notably, NAFLD patients have a significantly higher mortality risk than individuals without NAFLD; even in patients with mild fatty liver, the risk of death increased by 71% (8). Given all these considerations, NAFLD is now emerging as a public health challenge. A convenient and useful indicator would greatly facilitate the risk appraisal and subsequent prevention of NAFLD.

The prevailing hypothesis termed “multiple parallel hits” explains the pathogenesis of NAFLD from different perspectives, in which insulin resistance, oxidative stress, and inflammatory response play important roles (9). Red blood cell (RBC) count is a readily available, inexpensive hematological index in routine clinical examinations. In addition to the well-known function of sustaining aerobic respiration by transporting oxygen and carbon dioxide, red blood cells (RBCs) also play a part in modulating inflammation, immune responses, and oxidative stress (10), suggesting that RBCs may be a sensitive index reflecting certain conditions of the body. RBCs should not be ignored in the exploration of many disease processes (11). An increasing number of investigators have noted that a high RBC count might indicate an increased risk of metabolic syndrome (MS), insulin resistance, or hyperinsulinemia (12–14), which have an inseparable relationship with NAFLD. Moreover, our previous lipidomics study provided some information about the relationship between NAFLD and RBC (11).

Nevertheless, the longitudinal association between RBC count and NAFLD is unknown. Therefore, we conducted a large-scale longitudinal cohort study based on health assessments conducted in a Chinese urban population to evaluate the association of RBC count with the incidence (including severity) and progression of NAFLD. Our findings suggested that RBC count was positively associated with the risk for incident NAFLD and NAFLD progression. The findings may assist in identifying a convenient and useful indicator to use for further NAFLD risk appraisal and may provide novel insights into underlying NAFLD mechanisms as well as possible novel interventions for NAFLD.

## Materials and Methods

### Study Population

**Figure 1:**
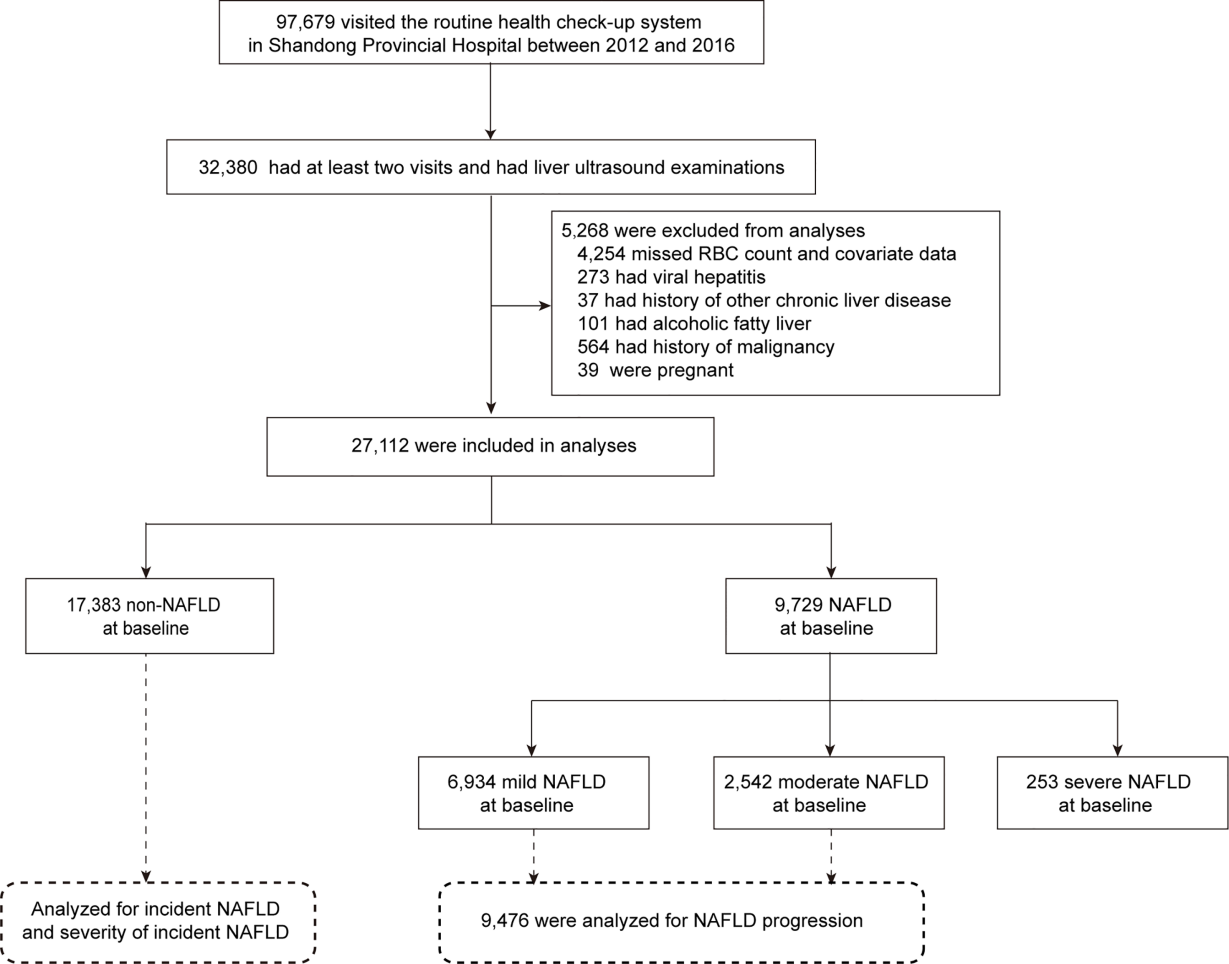
Participant flow diagram.

The study population was derived from a routine health check-up system based in the Health Management Center of Shandong Provincial Hospital between 2012 and 2016 (n=97,679). We recruited 32,380 subjects who had at least two visits and underwent liver ultrasound examinations during this period. Participants with the following conditions during the follow-up period were further excluded: 1) missing RBC data and missing covariate data such as sex, age, body mass index (BMI), systolic blood pressure (SBP), triglycerides (TGs), high-density lipoprotein cholesterol (HDL-C), low-density lipoprotein cholesterol (LDL-C), fasting plasma glucose (FPG), hemoglobin (Hb), smoking status, exercise, and dietary habits because the generalized estimation equation (GEE) method we used in this study does not allow for any missing values; 2) diagnosis of viral hepatitis; 3) a history of other chronic liver diseases such as autoimmune hepatitis; 4) alcoholic fatty liver; and 5) conditions during the study period that affect liver status or RBCs, such as pregnancy and a history of malignancy. Ultimately, a total of 27,112 subjects were eligible. Among them, 17,383 subjects without NAFLD at baseline were analyzed for the incidence of NAFLD, including incident NAFLD and the severity of incident NAFLD. A total of 9,476 subjects with mild or moderate NAFLD at baseline were analyzed for NAFLD progression (**Figure 1**). The number of health check-up system visits of 27,112 subjects is shown in **Supplemental Figure S1**. This study was approved by the Ethics Committee of Shandong Provincial Hospital affiliated with Shandong University (LCYJ: NO. 2019-002).

### Medical Measurements

The study visits took place in the morning at Shandong Provincial Hospital. All subjects completed a standardized questionnaire, including information on medical history and lifestyle (such as smoking status, exercise, and diet habits), which was administered by trained interviewers. Clinical assessments, including height, weight, blood pressure (BP), and liver ultrasound, were performed by experienced physicians in the morning. Weight and height were measured with electronic scales. BMI was calculated as weight (kg)/height squared (m^2^). After 5-15 minutes of rest, two BP values were taken on the right arm, and the mean value was reported. After an overnight fast of at least 10 hours, each subject provided blood samples from the antecubital vein. RBC count and Hb were quantified using an automated blood cell counter (XN-1000, Sysmex, Kobe, Japan). Other laboratory measurements included FPG, TGs, total cholesterol (TC), LDL-C, HDL-C, aspartate aminotransferase (AST), alanine aminotransferase (ALT), and hepatitis B virus testing.

### Diagnosis of NAFLD and Severity of NAFLD

The diagnosis of fatty liver and its severity were determined by professional sonographers using ultrasonography (LOGIQ P6, GE Ultrasound, Korea). The sonographers were blinded to the study design and any relevant laboratory information. Fatty liver status was defined as bright echoes in the liver, ultrasonic attenuation in a deep area of the liver, and vascular blurring (15, 16). According to the echogenicity of the liver parenchyma, fatty liver was classified into three grades: mild, moderate, and severe. Mild fatty liver refers to a slight, diffuse increase in echogenicity of the liver parenchyma and no obvious or slight attenuation of far field ultrasound echo with normal visualization of the diaphragm and intrahepatic vessel boundaries. Moderate fatty liver refers to a moderate diffuse increase in echogenicity of the liver parenchyma and moderate attenuation of far field ultrasound echo with slightly impaired visualization of the diaphragm and intrahepatic vessel boundaries. Severe fatty liver refers to an apparent increase in echogenicity of the liver parenchyma and obvious attenuation of far field ultrasound echo with marked impaired visualization or nonvisualization of the diaphragm and intrahepatic vessel boundaries (15, 17). Representative images are in **Supplemental Figure S2**. According to the diagnostic criteria of NAFLD formulated by the Chinese Society of Hepatology, fatty liver confirmed by ultrasonic imaging was considered to indicate NAFLD in this study unless the following conditions existed: 1) diagnosis of viral hepatitis; 2) a history of other chronic liver diseases such as autoimmune hepatitis; and 3) AST/ALT ratio > 2 and regular drinking, which is a surrogate estimate of alcoholic fatty liver (18, 19).

Incident NAFLD was defined as having no baseline NAFLD but presenting NAFLD during follow-up, and NAFLD progression was defined as an increase of at least one level in severity grade during follow-up in individuals with a NAFLD severity level of mild or moderate at baseline.

### Statistical Analyses

Continuous variables with normal or skewed distribution are presented as the mean (standard deviation) or median (interquartile range), respectively. Differences in characteristics between groups were analyzed using Student’s t-test, Mann-Whitney U test, one-way ANOVA, and Kruskal–Wallis H test for continuous variables. All *post hoc* tests were Bonferroni corrected. Categorical variables are presented as a number (percent). The chi-square test was used to assess the differences in categorical data. A linear trend chi-square test was conducted to assess the trends of categorical variables. Quartiles of RBC count during the entire study follow-up period were defined by sex-specific cutoffs. We calculated the incidence density (95% CI) per 100 person-years for each RBC quartile. Incident density is a measure of incidence that incorporates time directly into the denominator. Incident density = number of new cases/total number of observed person-years×100.

Binary and ordinal generalized estimation equation (GEE) methods, the extension of generalized linear models that allow for analysis of repeated measurements (20), were used to investigate the association between RBC count and NAFLD. To demonstrate the independent association between RBC count and the risk for incident NAFLD and NAFLD progression, Hb concentration was adjusted in GEE models. Given the multicollinearity between RBC count and Hb concentrations, we adjusted for the residual errors of regressing Hb on RBC count. Other potential confounders, including age, sex, BMI, SBP, FPG, TGs, HDL-C, LDL-C, diet, smoking, and exercise, were all adjusted in the multivariate GEE. Considering the temporality between exposures and outcomes, follow-up time was also adjusted.

We conducted two further sensitivity analyses. First, missing values were imputed using multiple imputation by chained equations, and we repeated all analyses using the imputed data set (19802 NAFLD and 10702 mild or moderate NAFLD at baseline). Second, we further adjusted for white blood cell (WBC) count and ALT on the basis of Model 2. A two-tailed P-value < 0.05 was considered statistically significant. The association of RBC count with the severity of incident NAFLD was assessed by SPSS (version 25.0) using ordinal GEE. All other statistical analyses were performed with R (version 4.2.0).

## Results

### Characteristics of Study Participants

Among 32,380 individuals who attended at least two visits and underwent liver ultrasound examinations between 2012 and 2016, a total of 27,112 subjects (17,367 men and 9,745 women) were included in the final analysis, with 9,729 (35.9%) confirmed as having NAFLD upon ultrasound (**Figure 1**). A total of 27,112 subjects contributed 85,062 observations (**Supplemental Figure S1**). **Table 1** presents the baseline characteristics according to NAFLD status. Compared with subjects without NAFLD, those with NAFLD were older; had a higher proportion of men, meatatarians and smokers; had a lower proportion of vegetarians and nonsmokers; and had a significantly higher BMI, SBP, FPG, TGs, TC, LDL-C, AST, and ALT and lower HDL-C (all P<0.001). Moreover, RBC count and Hb were higher in the NAFLD group than in the non-NAFLD group (P<0.001). Subjects with NAFLD were further divided into three groups according to severity: mild NAFLD (n = 6,934), moderate (n = 2,542), and severe NAFLD groups (n = 253). Interestingly, subjects with moderate and severe NAFLD displayed significantly higher RBC counts than individuals with mild NAFLD (P<0.001). **Supplemental Table S1** compares the baseline characteristics of participants according to sex. All of the above variables were significantly different between men and women (all P<0.001), and NAFLD in men was more serious (P<0.001).

**Table 1:** Baseline characteristics of study subjects based on NAFLD status.

Characteristic	NAFLD or Not			Degree of NAFLD			
	Non-NAFLD	NAFLD	P Value	Mild NAFLD	Moderate NAFLD	Severe NAFLD	P Value
Participants (%)	17,383 (64.1)	9729 (35.9)		6934 (71.3)	2542 (26.1)	253 (2.6)	
Age, y	42 (21)	47 (18)	<0.001	48 (18)	46 (17)	43 (22)	<0.001
Sex (%)			<0.001				<0.001
Men	9310 (53.6)	8057 (82.8)		5683 (82.0)	2153 (84.7)	221 (87.4)	
Women	8073 (46.4)	1672 (17.2)		1251 (18.0)	389 (15.3)	32 (12.6)	
RBC, cells×10^12^/L	4.80 ± 0.45	5.07 ± 0.40	<0.001	5.05 ± 0.40	5.12 ± 0.39 ^†^	5.13 ± 0.44 ^†^	<0.001
Hb, g/L	146 (23)	156 (16)	<0.001	156 (16)	158 (15) ^†^	157 (14)	<0.001
Diet (%)			<0.001				<0.001
Vegetarian	3579 (20.6)	1317 (13.5)		996 (14.4)	298 (11.7)	23 (9.1)	
Dishes mix	12,583 (72.4)	7111 (73.1)		5096 (73.5)	1838 (72.3)	177 (70.0)	
Meatatarian	1221 (7.0)	1301 (13.4)		842 (12.1)	406 (16.0)	53 (20.9)	
Smoking (%)			<0.001				<0.001
Non-smoker	13,997 (80.5)	6298 (64.7)		4503 (64.9)	1634 (64.3)	161 (63.6)	
Smoker	3386 (19.5)	3431 (35.3)		2431 (35.1)	908 (35.7)	92 (26.4)	
Exercise (%)			0.466				<0.001
Occasionally	12,687 (73.0)	7070 (72.6)		4930 (71.1)	1920 (75.5)	210 (83.0)	
Regularly	4696 (27.0)	2669 (27.4)		2004 (28.9)	622 (24.5)	43 (17.0)	
BMI, kg/m2	23.36 ± 2.93	27.06 ± 2.88	<0.001	26.53 ± 2.60	28.18 ± 3.00 ^†^	30.41 ± 3.54 ^† ‡^	<0.001
SBP, mmHg	116 (24)	128 (22)	<0.001	127 (22)	131 (21) ^†^	135(21) ^† ‡^	<0.001
FPG, mmol/L	5.10 (0.72)	5.44 (1.04)	<0.001	5.38 (0.93)	5.62 (1.22) ^†^	5.73 (1.96) ^† ‡^	<0.001
TG, mmol/L	0.99 (0.65)	1.69 (1.17)	<0.001	1.60 (1.08)	1.94 (1.41) ^†^	2.21 (1.52) ^†^	<0.001
TC, mmol/L	5.00 ± 0.92	5.36 ± 0.97	<0.001	5.32 ± 0.95	5.45 ± 0.99 ^†^	5.46 ± 1.03	<0.001
HDL-C, mmol/L	1.43 ± 0.34	1.21 ± 0.26	<0.001	1.23 ± 0.26	1.17 ± 0.25 ^†^	1.13 ± 0.23 ^†^	<0.001
LDL-C, mmol/L	2.86 ± 0.79	3.19 ± 0.84	<0.001	3.17 ± 0.82	3.23 ± 0.87 ^†^	3.24 ± 0.88	<0.001
AST, IU/L	20 (6)	23 (7)	<0.001	22 (7)	25 (12) ^†^	29 (14) ^† ‡^	<0.001
ALT, IU/L	17 (10)	26 (17)	<0.001	24 (15)	33 (25) ^†^	47 (36) ^† ‡^	<0.001

NAFLD, non-alcoholic fatty liver disease; Non-NAFLD, without NAFLD; y, years; RBC, red blood cell; Hb, hemoglobin; BMI, body mass index; SBP, systolic blood pressure; FPG, fasting plasma glucose; TG, triglyceride; TC, total cholesterol; HDL-C, high-density lipoprotein cholesterol; LDL-C, low-density lipoprotein cholesterol; AST, aspartate aminotransferase; ALT, alanine aminotransferase.

Data with normal distributions are reported using mean ± standard deviation; data with nonnormal distributions are reported using median (interquartile range); data with categorical variables are reported using number (percent). ^†^P < 0.05 vs mild NAFLD; ^‡^P < 0.05 vs moderate NAFLD.

**Figure 2:**
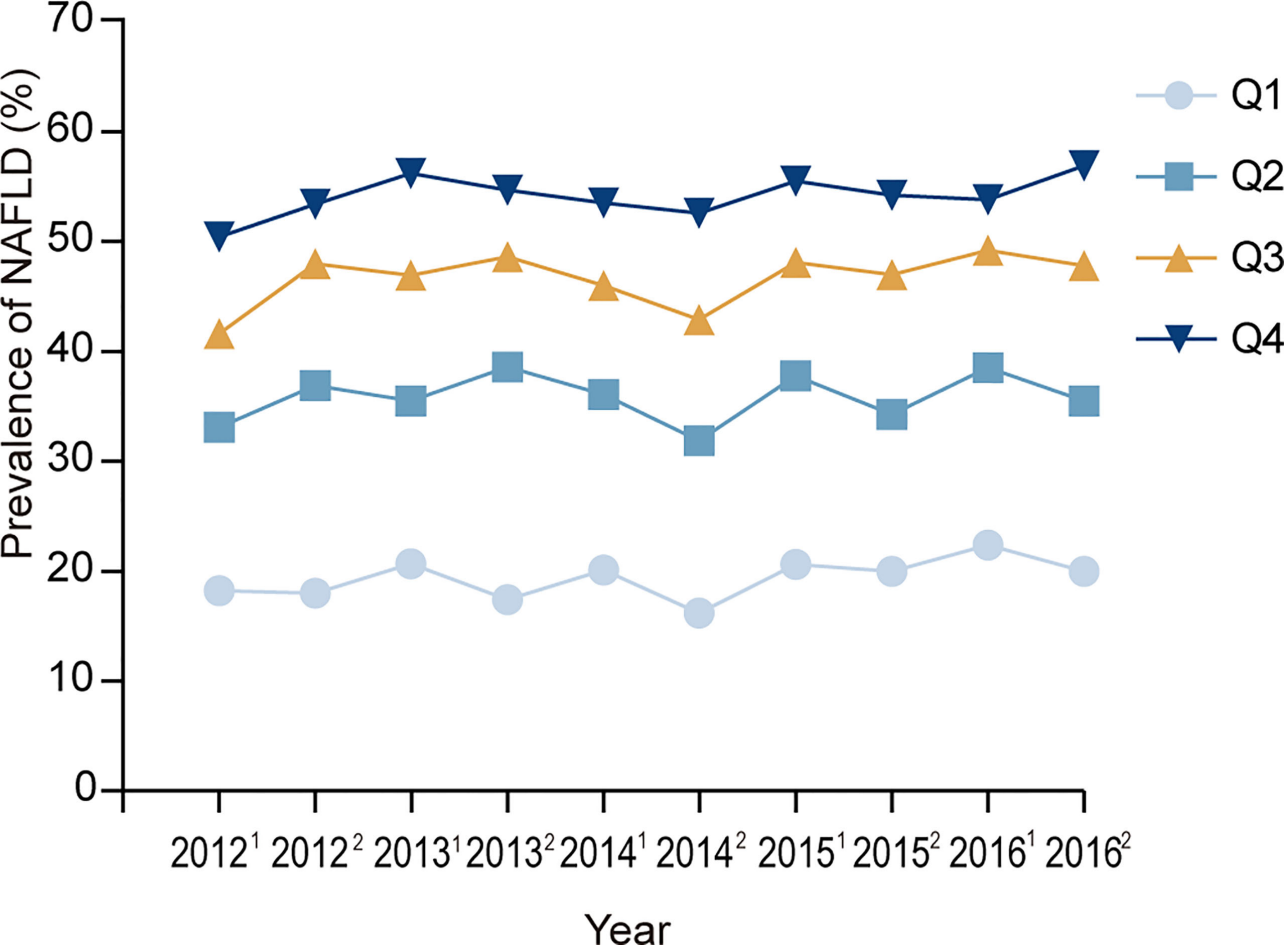
Prevalence of NAFLD at each visit time point. 2012^1^, 2012^2^, 2013^1^,2013^2^, ……2016^1^,2016^2^ represent the first half of 2012, the second half of 2012, the first half of 2013, the second half of 2013, …… the first half of 2016, and the second half of 2016. Q1, Q2, Q3, Q4 represent the lowest quartile, second quartile, third quartile, highest quartile of RBC count respectively.

Because some subjects received health examinations once every six months, we calculated the half-year prevalence of NAFLD. **Figure 2** presents the half-year prevalence of NAFLD according to RBC count quartile. During the entire follow-up period, the prevalence of NAFLD always increased with the increase in the quartile of RBC count.

### The Increased Risk for Incident NAFLD and Higher NAFLD Severity Category Associated With Higher RBC Count

**Figure 3:**
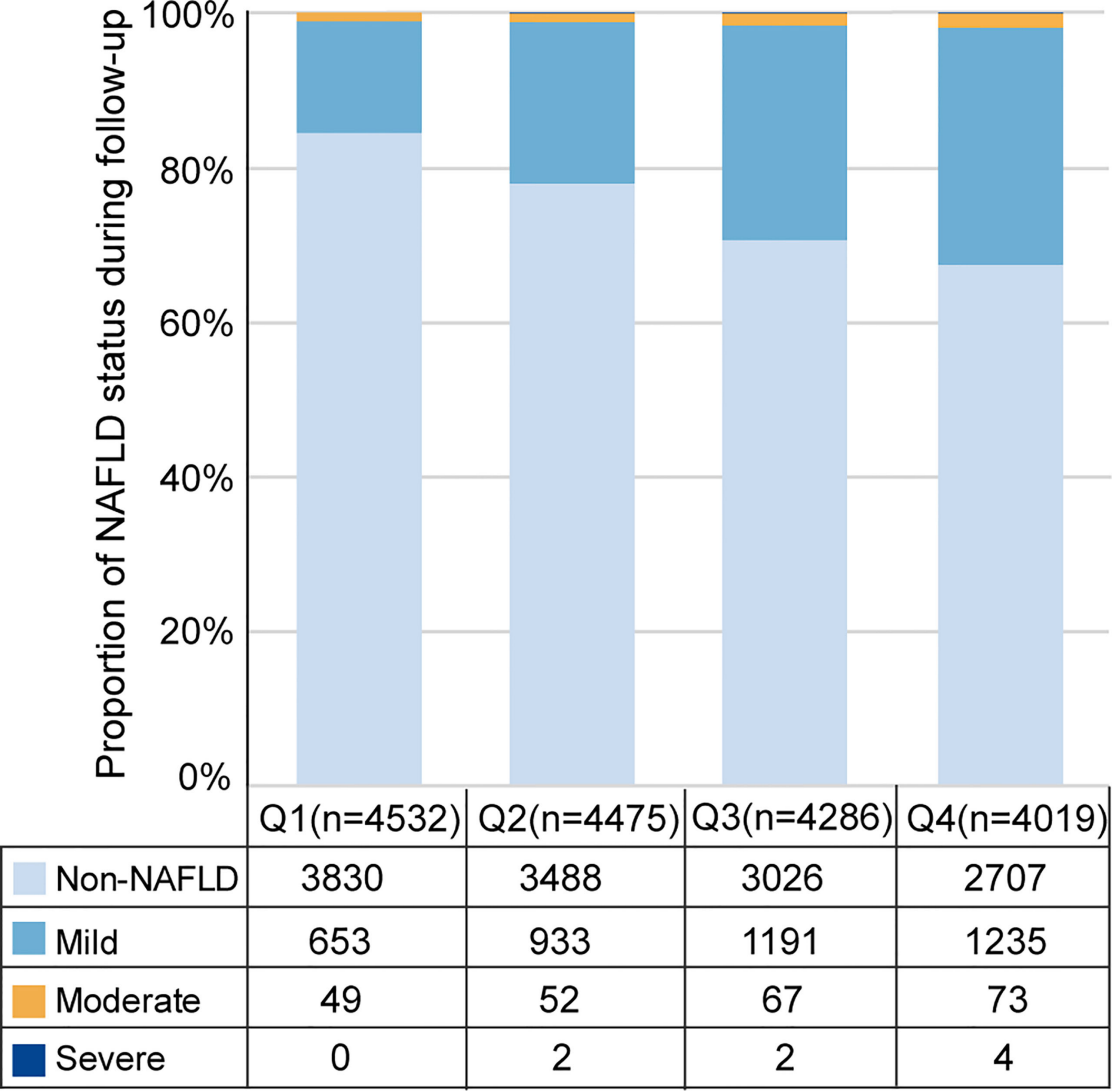
Distribution of incident NAFLD cases and different severity of incident NAFLD cases over RBC count quartiles. Q1, Q2, Q3, Q4 represent the lowest quartile, second quartile, third quartile, highest quartile of RBC count respectively.

During up to 5 years of follow-up (median 2.8 years), 4,332 (24.9%) incident cases of NAFLD were identified among 17,383 subjects without NAFLD at baseline. Among the cases of NAFLD, 4,012 (23.1%) were classified as mild, 241 (1.4%) were classified moderate, 8 (0.05%) were classified severe, and 71 were not classified. The distribution of incident NAFLD cases and different severities of incident NAFLD cases according to RBC count quartiles is illustrated in **Figure 3**. Incident NAFLD was detected in 32.6% of subjects in fourth quartile (Q4), while 18.3% in lowest quartile (Q1), with a consecutive increase over the quartiles. When focusing on the three severity categories, an intracategorical increase was seen in each of the three RBC count quartiles (second quartile (Q2), third quartile (Q3), and Q4). Linear trend Chi-square test showed that the severity of NAFLD had a tendency to increase as RBC count quartiles increased (r=0.343, P<0.001).

**Figure 4:**
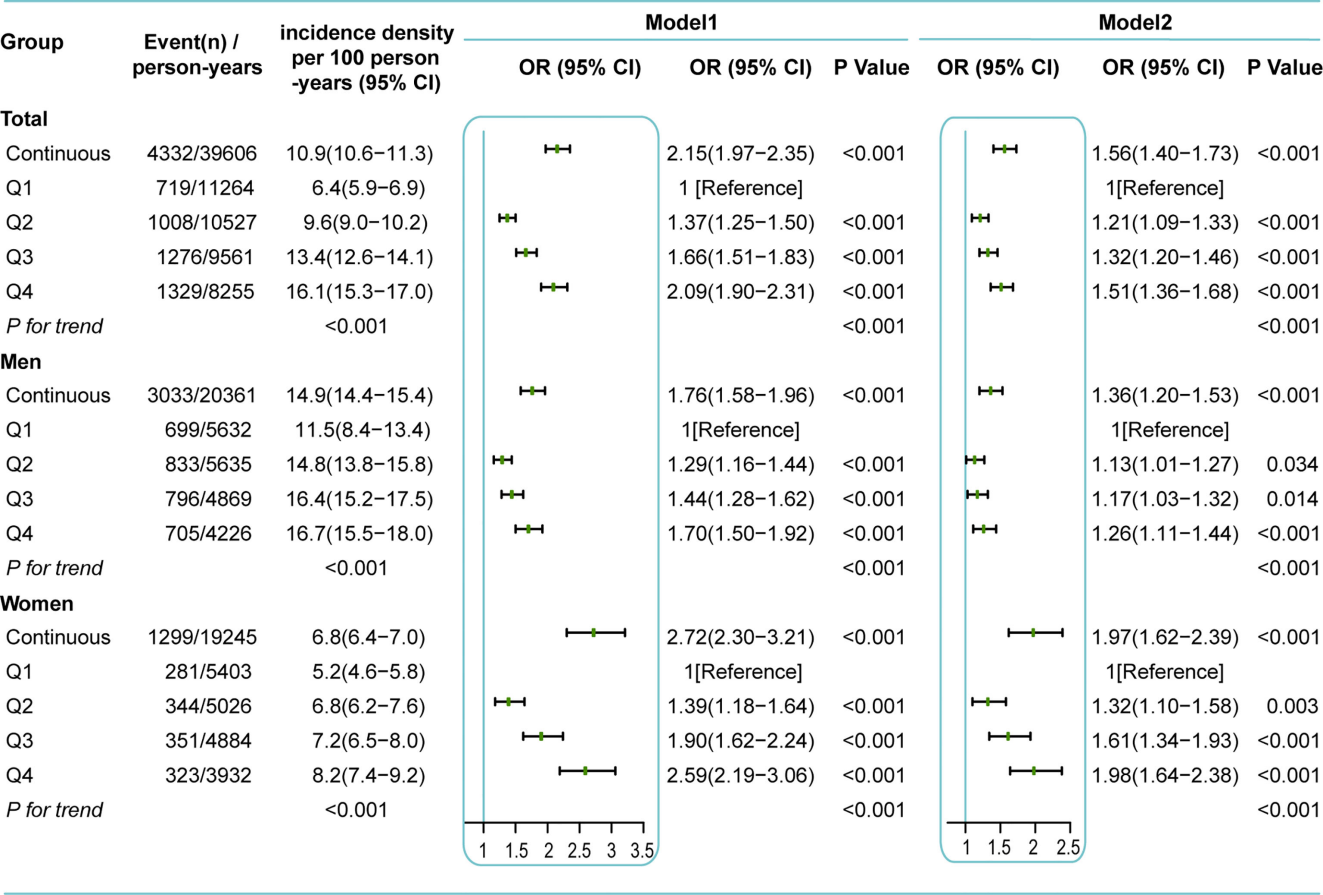
Incidence density and GEE analysis for incident NAFLD based on RBC count during follow-up. Q1, the lowest quartile of RBC count (<4.83×10^12^cells/L for men, <4.33×10^12^cells/L for women); Q2, the second quartile of RBC count (4.83-5.08×10^12^cells/L for men, 4.33-4.52×10^12^cells/L for women); Q3, the third quartile of RBC count (5.09-5.32×10^12^cells/L for men, 4.53-4.74×10^12^cells/L for women); Q4, the highest quartile of RBC count (>5.32×10^12^cells/L for men, >4.74×10^12^cells/L for women). Model 1 was adjusted for age, follow-up time. Model 2 was adjusted for age, BMI, SBP, GLU, TG, HDL-C, LDL-C, diet, smoking, exercise, follow-up time, and residual errors of regressing Hb on RBC. Sex was also adjusted in all models of the total group.

The incidence density of NAFLD also tended to increase along as RBC count quartiles increased; the incidence densities of NAFLD were 6.4, 9.6, 13.4, and 16.1/100 person-years, respectively (P for trend < 0.001) (**Figure 4**). A similar trend was observed in both men and women, while the incidence density was significantly higher in men than in women (14.9 *vs* 6.8/100 person-years). Age-, sex- and follow-up time-adjusted GEE analysis showed that the risk of incident NAFLD increased by 2.15-fold (OR 2.15 [95% CI 1.97–2.35]) for every one-unit (10^12^ cells/L) increase in RBC count. When RBC count was considered a categorical variable, there was a dose-dependent positive correlation between RBC count and the risk of incident NAFLD (P for trend < 0.001). Compared with Q1, the ORs and 95% CIs of the other RBC count quartiles (Q2, Q3, Q4) were 1.37 (1.25-1.50), 1.66 (1.51-1.83), and 2.09 (1.90-2.31), respectively (Model 1, **Figure 4**). Further adjustment for confounders (age, sex, BMI, SBP, GLU, TGs, HDL-C, LDL-C, Hb, smoking, exercise, diet, follow-up time and residual errors of regressing Hb on RBC count) did not markedly alter the observed associations; the adjusted ORs and 95% CIs of Q2, Q3, and Q4 *vs* Q1 were 1.21 (1.09-1.33), 1.32 (1.20-1.46), and 1.51 (1.36-1.68) (P for trend < 0.001), respectively. Every one-unit (10^12^ cells/L) increase in RBC count was accompanied by a 1.56-fold increase in incident NAFLD (OR, 1.56; 95% CI, 1.40–1.73) (Model 2, **Figure 4**). The positive associations between RBC count and incident NAFLD were consistent in both men and women but appeared to be more pronounced in women (**Figure 4**).

To assess the association between RBC count and NAFLD in better detail, we next studied the severity of incident NAFLD as an outcome. Ordinal GEE analytical results showed a close association between RBC count and NAFLD severity. In the fully adjusted models, every one-unit (10^12^ cells/L) increase in RBC count was associated with a 51% increased risk of falling into a higher NAFLD severity category (OR=1.51 [95% CI, 1.36–1.68]). When introducing RBC count as an ordered categorical variable, Q2 [OR=1.43 (95% CI, 1.28–1.60)], Q3 [OR=1.57 (95% CI, 1.39–1.79)] and Q4 [OR=1.67 (95% CI, 1.45–1.93)] were still associated with a higher NAFLD severity category than Q1 (P for trend<0.001) (Model 2, **Table 2**). A stronger association could be seen in women than in men (**Table 2**).

**Table 2:** GEE analysis for severity of incident NAFLD based on RBC count during follow-up.

	Unadjusted		Model 1		Model 2	
	OR (95% CI)	P Value	OR (95% CI)	P Value	OR (95% CI)	P Value
**RBC in Total (10^12^ cells/L)**						
Continuous	2.53 (2.37-2.71)	<0.001	2.19 (1.05-2.00)	<0.001	1.51 (1.36-1.68)	<0.001
Q1	1 [Reference]		1 [Reference]		1 [Reference]	
Q2	1.78 (1.61-1.96)	<0.001	1.68 (1.51-1.87)	<0.001	1.43 (1.28-1.60)	<0.001
Q3	2.63 (2.38-2.91)	<0.001	2.21 (1.97-2.49)	<0.001	1.57 (1.39-1.79)	<0.001
Q4	3.35 (3.03-3.70)	<0.001	2.75 (2.43-3.12)	<0.001	1.67 (1.45-1.93)	<0.001
*P for trend*		<0.001		<0.001		<0.001
**RBC in Men (10^12^ cells/L)**						
Continuous	1.73 (1.57-1.90)	<0.001	1.77 (1.58-1.97)	<0.001	1.30 (1.15-1.48)	<0.001
Q1	1 [Reference]		1 [Reference]		1 [Reference]	
Q2	1.42 (1.18-1.71)	<0.001	1.60 (1.30-1.96)	<0.001	1.32 (1.07-1.63)	0.01
Q3	1.76 (1.47-2.12)	<0.001	1.98 (1.62-2.43)	<0.001	1.42 (1.15-1.76)	0.001
Q4	2.07 (1.73-2.49)	<0.001	2.35 (1.91-2.90)	<0.001	1.49 (1.20-1.85)	<0.001
*P for trend*		<0.001		<0.001		0.001
**RBC in Women (10^12^ cells/L)**						
Continuous	2.84 (2.41-3.35)	<0.001	2.82 (2.37-3.35)	<0.001	2.00 (1.64-2.44)	<0.001
Q1	1 [Reference]		1 [Reference]		1 [Reference]	
Q2	1.63 (1.44-1.83)	<0.001	1.73 (1.52-1.97)	<0.001	1.44 (1.25-1.66)	0.004
Q3	2.19 (1.89-2.54)	<0.001	2.25 (1.92-2.64)	<0.001	1.60 (1.34-1.91)	0.010
Q4	2.65 (2.09-3.36)	<0.001	2.53 (1.94-3.30)	<0.001	1.73 (1.29-2.32)	0.027
*P for trend*		<0.001		<0.001		<0.001

OR, odds ratio; 95% CI, 95% confidence interval; Q1, the lowest quartile of RBC count (<4.83×10^12^cells/L for men, <4.33×10^12^cells/L for women); Q2, the second quartile of RBC count (4.83-5.08×10^12^cells/L for men, 4.33-4.52×10^12^cells/L for women); Q3, the third quartile of RBC count (5.09-5.32×10^12^cells/L for men, 4.53-4.74×10^12^cells/L for women); Q4, the highest quartile of RBC count (>5.32×10^12^cells/L for men, >4.74×10^12^cells/L for women).

Model 1 was adjusted for age, follow-up time. Model 2 was adjusted for age, BMI, SBP, GLU, TG, HDL-C, LDL-C, diet, smoking, exercise, follow-up time, and residual errors of regressing Hb on RBC. Sex was also adjusted in Model 1 and Model 2 of the total group.

### The Association Between Higher RBC Count and Increased Risk for NAFLD Progression

As a continuous disease spectrum, NAFLD has the potential to lead to more serious and irreversible states, such as fibrosis and cirrhosis. Thus, we also focused on the association between RBC count and NAFLD progression. Finally, 1,798 of 9,476 (19.0%) participants with a mild or moderate NAFLD severity level at baseline experienced NAFLD progression during follow-up, including 1,658 with mild NAFLD progression and 140 with moderate NAFLD progression. The incidence density of NAFLD progression tended to increase as RBC count quartiles increased; the incidence densities were 7.6, 7.7, 8.0, and 9.4/100 person-years, respectively (P for trend<0.001). **Table 3** shows the results of GEE analysis for NAFLD progression based on RBC count during follow-up. Considering that the possibility of NAFLD progression may vary between mild and moderate groups, we further adjusted for the baseline severity grade of NAFLD in the final model. The risk of NAFLD progression increased progressively as RBC count increased, with adjusted ORs for Q2, Q3, and Q4 *vs* Q1 of 1.19 (1.03-1.37), 1.31 (1.13-1.51), and 1.52 (1.31-1.77), respectively (P for trend = 0.001). Every one-unit (10^12^ cells/L) increase in RBC count was associated with a 53% increased risk for NAFLD progression [OR 1.53 (95% CI 1.32-1.77)]. The sex-specific results showed a similar pattern (Model 2, **Table 3**).

**Table 3:** GEE analysis for NAFLD progression based on RBC count during follow-up.

	Unadjusted		Model 1		Model 2	
	OR (95% CI)	P Value	OR (95% CI)	P Value	OR (95% CI)	P Value
**RBC in Total (10^12^ cells/L)**						
Continuous	1.58 (1.40-1.77)	<0.001	1.72 (1.51-1.96)	<0.001	1.53 (1.32-1.77)	<0.001
Q1	1 [Reference]		1 [Reference]		1 [Reference]	
Q2	1.25 (1.09-1.44)	0.002	1.26 (1.10-1.45)	<0.001	1.19 (1.03-1.37)	0.016
Q3	1.44 (1.25-1.66)	<0.001	1.42 (1.23-1.64)	<0.001	1.31 (1.13-1.51)	<0.001
Q4	1.83 (1.59-2.10)	<0.001	1.72 (1.49-1.98)	<0.001	1.52 (1.31-1.77)	<0.001
* P for trend*		<0.001		<0.001		0.001
**RBC in Men (10^12^ cells/L)**						
Continuous	1.74 (1.51-2.00)	<0.001	1.59 (1.37-1.84)	<0.001	1.41 (1.20-1.66)	<0.001
Q1	1 [Reference]		1 [Reference]		1 [Reference]	
Q2	1.20 (1.03-1.40)	0.018	1.20 (1.03-1.40)	0.019	1.14 (0.98-1.34)	0.090
Q3	1.41 (1.20-1.64)	<0.001	1.37 (1.17-1.61)	<0.001	1.27 (1.08-1.49)	0.005
Q4	1.75 (1.51-2.05)	<0.001	1.62 (1.39-1.90)	<0.001	1.45 (1.23-1.72)	<0.001
* P for trend*		<0.001		<0.001		0.001
**RBC in Women (10^12^ cells/L)**						
Continuous	2.51 (1.83-3.44)	<0.001	2.30 (1.66-3.17)	<0.001	2.10 (1.48-2.96)	0.081
Q1	1 [Reference]		1 [Reference]		1 [Reference]	
Q2	1.46 (1.06-2.03)	0.022	1.47 (1.08-2.02)	0.017	1.38 (1.00-1.91)	0.048
Q3	1.57 (1.14-2.15)	0.006	1.51 (1.10-2.08)	0.011	1.41 (1.02-1.96)	0.037
Q4	2.12 (1.54-2.91)	<0.001	1.96 (1.42-2.71)	<0.001	1.72 (1.24-2.40)	0.001
* P for trend*		<0.001		<0.001		0.002

OR, odds ratio; 95% CI, 95% confidence interval; Q1, the lowest quartile of RBC count (<4.92×10^12^cells/L for men, <4.49×10^12^cells/L for women); Q2, the second quartile of RBC count (4.92-5.16×10^12^cells/L for men, 4.49-4.67×10^12^cells/L for women); Q3, the third quartile of RBC count (5.17-5.40×10^12^cells/L for men, 4.68-4.88×10^12^cells/L for women); Q4, the highest quartile of RBC count (>5.40×10^12^cells/L for men, >4.88×10^12^cells/L for women).

Model 1 was adjusted for adjustment for age, follow-up time. Model 2 was adjusted for age, BMI, SBP, GLU, TG, HDL-C, LDL-C, Hb, diet, smoking, exercise, follow-up time, severity of NAFLD at baseline, and residual errors of regressing Hb on RBC. Sex was also adjusted in Model 1 and Model 2 of the total group.

### Sensitivity Analyses

The associations between RBC count and NAFLD (including incident, severity, and progression) were similar when imputed data analyses were conducted (**Supplemental Tables S2**, **S3**). The results did not significantly change after further adjusting for WBC count and ALT on the basis of Model 2 (**Supplemental Table S4**).

## Discussion

This is the first population-based large longitudinal cohort study to investigate the association between RBC count and NAFLD (incident, severity, and progression) and suggests that increased RBC count is a previously unrecognized risk factor for the occurrence and progression of NAFLD. During the 5-year follow-up period, RBC count was positively associated with incident NAFLD and the severity of incident NAFLD independent of Hb and the other traditional risk factors for NAFLD. Furthermore, we demonstrated that a higher RBC count was associated with a higher risk of NAFLD progression. These findings may provide evidence in support of using readily available, inexpensive, routinely collected clinical erythrocytic parameters for further risk appraisal of NAFLD. Individuals with high RBC counts may require early screening and prevention of NAFLD incidence and progression. In addition, our findings may facilitate the understanding of the underlying mechanisms of NAFLD as well as therapeutic strategies for this disease.

A limited number of studies have explored the relationship between RBC count and fatty liver. Consistent with our findings, a cross-sectional study found that elevated RBC count was independently associated with a high risk of developing fatty liver (21), but the study did not rule out the effects of alcohol consumption. Another cross-sectional study by Jiang Y et al. also demonstrated the positive correlation between RBC count and fatty liver index (22), which showed a good ability to distinguish individuals with NAFLD from those without it (23). A study on pediatric populations reported that the RBC count was significantly higher in the biopsy-diagnosed nonalcoholic steatohepatitis (NASH) group than in the nonalcoholic fatty liver (NAFL) group (5.00×10^12^/L *vs* 5.29 ×10^12^/L, P=0.01) (24), suggesting that the RBC count might be related to NAFLD progression. However, the above studies could not confirm whether increased RBC count precedes the onset of NAFLD due to the cross-sectional nature. And they focused only on incident NAFLD and a simple comparison of RBC counts among individuals with different NAFLD severities. These studies do not answer the question of the temporal relationship between RBC count and NAFLD in detail. In the present study, we employed a longitudinal cohort design and a larger natural population. We found that RBC count was correlated not only with incident NAFLD but also with the severity of incident NAFLD. Importantly, we are the first to explore the relationship between RBC count and NAFLD progression. NAFLD is a continuous disease spectrum, potentially leading to more serious and irreversible states, such as fibrosis and cirrhosis. Consequently, it is very important to identify the population at high risk of NAFLD progression and carry out preventive measures. In addition, everyone in our study contributed multiple observations of RBC count, liver ultrasound, and other important indicators during follow-up. To make full use of each observation at each time point, we used the GEE method, which could not only accommodate the autocorrelation caused by repeated measurement but also avoid the contingency of the results caused by only two time points, thus improving the accuracy and credibility of the results (20). Therefore, the careful study design, higher number of subjects, and more accurate statistical methods used may improve the power of this study and provide the most reliable and up-to-date information on the relationship between RBC count and NAFLD in the adult Chinese population.

It is worth noting that Hb is a predominant component of RBCs and is the main function bearer of RBC function. Previous studies have revealed the association between increased Hb levels and a higher risk of NAFLD (22, 25, 26). We observed a similar phenomenon in this study (not listed). This phenomenon can be explained by iron overload, which is considered one of the essential factors causing NAFLD (27, 28). Previous studies on the relationship between RBCs and fatty liver did not evaluate the effect of Hb. Considering the close correlation between RBC count and Hb concentration, without adjusting for Hb, we could not determine whether the association between RBC count and NAFLD was real or just a false positive correlation secondary to the effect of Hb concentration. To solve this problem, we analyzed the independent association between RBC count and NAFLD, adjusting for not only common confounders but also Hb. Our results showed a significant positive association between RBC count and the incidence and progression of NAFLD in GEE analysis, even after adjusting for Hb and other confounding factors. This finding indicates that RBC count *per se* has an independent role in the occurrence and progression of NAFLD.

We can propose several potential mechanisms linking RBC count to NAFLD independent of Hb. First, we hypothesized that some substances secreted by RBCs may be involved in the occurrence and progression of NAFLD. Recent investigations clearly identified RBCs as a main source of sphingosine 1-phosphate (S1P) in plasma (29). Sphingosine can be incorporated into RBCs and then transformed into S1P, and a large amount of S1P in RBCs may leak out even without stimulation of RBCs (30). Naturally, an increase in RBC count resulted in an increase in the concentration of S1P released into the blood (31). S1P is now emerging as a factor involved in liver pathobiology, including NAFLD (32). Studies have reported that S1P in the extracellular environment binds to S1PR2 on hepatocytes, which results in diminished insulin signaling and insulin resistance (IR) (33). Chen et al. observed that treatment with S1P significantly enhanced hepatic lipid storage (34). In addition, we speculate that erythrocyte-derived microvesicles, which have been proven to promote cardiovascular disease by mediating inflammation (35), may also take part in the pathogenesis of NAFLD. However, the exact and detailed mechanism still needs further study. Second, RBC count may be related to insulin resistance and NAFLD by influencing blood viscosity (36). Because the count of RBCs is an important factor related to blood viscosity (37), blood viscosity increases with increasing RBC count (38). Higher blood viscosity could decrease blood flow and subsequently decrease the circulation of oxygen, glucose and insulin to essential tissues such as skeletal muscle (39, 40). The body will then increase blood flow through vasodilation, blood pressure elevation, and other compensatory mechanisms. However, when these mechanisms fail, an increase in glucose and insulin would be necessary to further increase their transport to skeletal muscle, which can cause insulin resistance (41, 42). In addition, reduced oxygen delivery leads to insufficient oxidation capacity. Increasing evidence has shown that insufficient oxidative capacity of muscle is the main cause of insulin resistance (43). Third, the erythrocyte membrane lipid profile may be another factor linking RBC count with NAFLD. Our previous study found that NAFLD was accompanied by changes in the composition of the erythrocyte membrane lipid profile (11).

Considering that the limits of normal RBC count in women differ from those in men, we conducted a subgroup analysis by sex. The sex-specific result showed that the association between RBC count and NAFLD was stronger for women than for men, suggesting that sex modified the effect between RBC count and NAFLD. At present, we have no clear explanation for the difference in the relationship between RBC count and NAFLD by sex. Perhaps erythrocytes in women are more fragile (44) and are more frequently engulfed by hepatic macrophages than those in males, resulting in more iron release. In addition, we hypothesize that sex hormones may regulate the relationship between the RBC count and NAFLD. However, we did not obtain data on sex hormones. Thus, further investigations are now needed to clarify the reasons for the above differences between men and women.

The strengths of the study are as follows: a longitudinal cohort design with a relatively large sample size was used, which allowed for more convincing conclusions; more accurate statistical methods, GEE methods, was used to improve the credibility of the conclusion; adjustment for Hb concentration was conducted for a rational evaluation of the independent effects of RBC count on NAFLD; and more detailed research content, including classification of NAFLD degree and NAFLD progression assessment, was used to obtain more comprehensive conclusions. Certain potential limitations exist in our study. First, the population may not be representative of the general population, as the subjects were recruited from a routine health check-up system in an urban Chinese population in Shandong Province. Second, NAFLD was diagnosed by ultrasound. However, ultrasonography is considered a first-line method for NAFLD diagnosis according to practical clinical guidelines (45), and it is particularly valuable for screening NAFLD high-risk groups in large populations given that it is impossible to obtain the gold-standard liver biopsy in apparently healthy subjects. Third, liver ultrasound scan was not performed by the same sonographer. However, sonographers in our study are experienced and professional.

In conclusion, our data demonstrate that elevated RBC count was independently associated with a high risk of incident NAFLD, severity of incident NAFLD, and NAFLD progression. High RBC count measured in the outpatient screening setting was identified as a risk factor for the development and progression of NAFLD in addition to other known risk factors. This finding may lead to a new interpretation of this routine clinical examination by primary care providers, thus increasing their attention to the increased risk of NAFLD among people who would usually not frequently be screened for this disease. Identifying individuals at higher risk for NAFLD may facilitate preventive interventions. Thus, individuals with increased RBC counts might require aggressive lifestyle modifications to prevent the occurrence and progression of NAFLD.

## Data Availability Statement

The datasets presented in this article are not readily available because the ethical approval obtained for this study prevents the human data being shared publicly to protect patients’ privacy. Requests to access the datasets would be passed to the ethics committee who will decide whether they can access the data directly. Requests to access the datasets should be directed to FZ, sdzf0404@163.com.

## Ethics Statement

The studies involving human participants were reviewed and approved by the Ethics Committee of Shandong Provincial Hospital affiliated with Shandong University. Written informed consent for participation was not required for this study in accordance with the national legislation and the institutional requirements.

## Author Contributions

FZ and MZ designed the study and wrote the manuscript. QL and YQ provided oversight to the study analyses. LG, LYG, and HL revised the manuscript and had final approval of the submitted and published versions. JZ is the guarantors of this work and, as such, had full access to all the data in the study and take responsibility for the integrity of the data and the accuracy of the data analysis. ZY and GY assisted to reply to the reviewer’s questions. All authors contributed to the article and approved the submitted version.

## Funding

This work was supported by grants from the National Key Research and Development Program of China (2017YFC1309800), and the National Natural Science Foundation (91957209).

## Conflict of Interest

The authors declare that the research was conducted in the absence of any commercial or financial relationships that could be construed as a potential conflict of interest.

## Publisher’s Note

All claims expressed in this article are solely those of the authors and do not necessarily represent those of their affiliated organizations, or those of the publisher, the editors and the reviewers. Any product that may be evaluated in this article, or claim that may be made by its manufacturer, is not guaranteed or endorsed by the publisher.
