# Supplementary material for: Red Blood Cell Count: An Unrecognized Risk Factor for Nonalcoholic Fatty Liver Disease
Source: Front Endocrinol (Lausanne). 2021 Dec 7;12:760981. doi: 10.3389/fendo.2021.760981 (PMC8688742; doi:10.3389/fendo.2021.760981)
Supplement: Supplementary file 1 [file DataSheet_1.docx]

**Supplemental Table S1** Baseline characteristics of study subjects based on gender

| Characteristic | Total | Men | Women | P Value |
| --- | --- | --- | --- | --- |
| Participants | 27,112 | 17,367 | 9745 |  |
| Age, y | 44 (21) | 46 (21) | 42.00 (20) | <0.001 |
| NAFLD, (%) | 9729 (35.9) | 8057 (46.4) | 1672 (17.2) | <0.001 |
| Mild | 6934 (25.6) | 5683 (70.5) | 1251 (74.8) |  |
| Moderate | 2542 (9.4) | 2153 (26.7) | 389 (23.3) |  |
| Severe | 253 (0.9) | 221 (2.7) | 32 (1.9) |  |
| RBC, cells×10^12^/L | 4.90 ± 0.45 | 5.10 ± 0.38 | 4.54 ± 0.32 | <0.001 |
| Hb, g/L | 151 (22) | 157(13) | 135 (13) | <0.001 |
| Diet (%) |  |  |  | <0.001 |
| Vegetarian | 4896 (18.1) | 2494 (14.4) | 2402 (24.6) |  |
| Dishes mix | 19,694 (72.6) | 12,786 (73.6) | 6908 (70.9) |  |
| Meatatarian | 2522 (9.3) | 2087 (12.0) | 435 (4.5) |  |
| Smoking (%) |  |  |  | <0.001 |
| Non-smoker | 20,295 (74.9) | 10,590 (61.0) | 9705 (99.6) |  |
| Smoker | 6817 (25.1) | 6777 (39.0) | 40 (0.4) |  |
| Exercise (%) |  |  |  | <0.001 |
| Occasionally | 19,747 (72.8) | 11,725 (67.5) | 8022 (82.3) |  |
| Regularly | 7365 (27.2) | 5642 (32.5) | 1723 (17.7) |  |
| BMI, kg/m^2^ | 24.69 ± 3.41 | 25.68 ± 3.09 | 22.91 ± 3.23) | <0.001 |
| SBP, mmHg | 121 (24) | 125 (22) | 112 (23) | <0.001 |
| FPG, mmol/L | 5.21 (0.84) | 5.30 (0.9) | 5.06 (0.69) | <0.001 |
| TG, mmol/L | 1.19 (0.93) | 1.37 (1.01) | 0.93 (0.67) | <0.001 |
| TC, mmol/L | 5.13 ± 0.95 | 5.15 ± 0.93 | 5.09 ± 0.99 | <0.001 |
| HDL-C, mmol/L | 1.35 ± 0.33 | 1.26 ± 0.28 | 1.52 ± 0.34 | <0.001 |
| LDL-C, mmol/L | 2.98 ± 0.82 | 3.03 ± 0.80 | 2.88 ± 0.86 | <0.001 |
| AST, IU/L | 21 (8) | 22 (8) | 19 (6) | <0.001 |
| ALT, IU/L | 20 (14) | 23 (15) | 15 (9) | <0.001 |

Abbreviations: y, years; RBC, red blood cell; Hb, hemoglobin; BMI, body mass index; SBP, systolic blood pressure; FPG, fasting plasma glucose; TG, triglyceride; TC, total cholesterol; HDL-C, high-density lipoprotein cholesterol; LDL-C, low-density lipoprotein cholesterol; AST, aspartate aminotransferase; ALT, alanine aminotransferase.

Data with normal distributions are reported using mean±standard; data with nonnormal distributions are reported using median (interquartile range); data with categorical variables are reported using number (percent).

**Supplemental Table S2** GEE analysis for incident NAFLD and severity of incident NAFLD based on RBC count during follow-up in participants with imputed data

|  | Unadjusted | | Model1 | | Model2 | |
| --- | --- | --- | --- | --- | --- | --- |
|  | OR (95% CI) | P Value | OR (95% CI) | P Value | OR (95% CI) | P Value |
| **Incident NAFLD** |  |  |  |  |  |  |
| Continuous | 2.49(2.33-2.66) | <0.001 | 2.20(2.03-2.39) | <0.001 | 1.64(1.49-1.80) | <0.001 |
| Q1 | 1 [Reference] |  | 1 [Reference] |  | 1 [Reference] |  |
| Q2 | 1.28(1.18-1.39) | <0.001 | 1.39(1.28-1.51) | <0.001 | 1.23(1.12-1.34) | <0.001 |
| Q3 | 1.52(1.40-1.65) | <0.001 | 1.68(1.54-1.83) | <0.001 | 1.35(1.23-1.48) | <0.001 |
| Q4 | 1.92(1.77-2.09) | <0.001 | 2.12(1.93-2.32) | <0.001 | 1.56(1.42-1.72) | <0.001 |
| *P for trend* |  | <0.001 |  | <0.001 |  | <0.001 |
| **Severity of**  **Incident NAFLD** |  |  |  |  |  |  |
| Continuous | 2.57 (2.41-2.74) | <0.001 | 2.24 (2.06-2.43) | <0.001 | 1.59 (1.44-1.75) | <0.001 |
| Q1 | 1 [Reference] |  | 1 [Reference] |  | 1 [Reference] |  |
| Q2 | 1.30 (1.19-1.40) | <0.001 | 1.46 (1.34-1.59) | <0.001 | 1.23 (1.12-1.34) | <0.001 |
| Q3 | 1.55 (1.43-1.68) | <0.001 | 1.76 (1.61-1.92) | <0.001 | 1.34 (1.23-1.48) | <0.001 |
| Q4 | 1.99(1.84-2.17) | <0.001 | 2.22 (2.03-2.44) | <0.001 | 1.54 (1.39-1.70) | <0.001 |
| *P for trend* |  | <0.001 |  | <0.001 |  | <0.001 |

Abbreviation: OR, odds ratio; 95% CI, 95% confidence interval.

Model 1 was adjusted for age, sex, and follow-up time. Model 2 was adjusted for age, sex, BMI, SBP, GLU, TG, HDL-C, LDL-C, diet, smoking, exercise, follow-up time, and residual errors of regressing Hb on RBC.

**Supplemental Table S3—GEE analysis for NAFLD progression based on RBC count during follow-up** **in participants with imputed data**

|  | Unadjusted | | Model1 | | Model2 | |
| --- | --- | --- | --- | --- | --- | --- |
|  | OR (95% CI) | P Value | OR (95% CI) | P Value | OR (95% CI) | P Value |
| **RBC count**  **(10^12^ cells/L)** |  |  |  |  |  |  |
| Continuous | 1.49(1.33-1.66) | <0.001 | 1.60(1.42-1.81) | <0.001 | 1.46(1.28-1.68) | <0.001 |
| Q1 | 1 [Reference] |  | 1 [Reference] |  | 1 [Reference] |  |
| Q2 | 1.18(1.04-1.34) | 0.009 | 1.19(1.05-1.35) | 0.005 | 1.14(1.00-1.29) | 0.045 |
| Q3 | 1.34(1.18-1.52) | <0.001 | 1.32(1.16-1.50) | <0.001 | 1.23(1.08-1.41) | 0.002 |
| Q4 | 1.71(1.50-1.93) | <0.001 | 1.61(1.42-1.84) | <0.001 | 1.46(1.27-1.67) | <0.001 |
| *P for trend* |  | <0.001 |  | <0.001 |  | <0.001 |

Abbreviation: OR, odds ratio; 95% CI, 95% confidence interval.

Model 1 was adjusted for age, sex, and follow-up time. Model 2 was adjusted for age, sex, BMI, SBP, GLU, TG, HDL-C, LDL-C, diet, smoking, exercise, follow-up time, and residual errors of regressing Hb on RBC.

**Supplemental Table S4—GEE analysis based on RBC count additionally adjusted for WBC and ALT during follow-up**

|  | **Incident NAFLD** | | **Severity of incident NAFLD** | | **NAFLD progression** | |
| --- | --- | --- | --- | --- | --- | --- |
|  | OR (95% CI) | P Value | OR (95% CI) | P Value | OR (95% CI) | P Value |
| **RBC count**  **(10^12^ cells/L)** |  |  |  |  |  |  |
| Continuous | 1.46(1.31-1.62) | <0.001 | 1.40(1.26-1.55) | <0.001 | 1.36(1.17-1.58) | <0.001 |
| Q1 | 1 [Reference] |  | 1 [Reference] |  | 1 [Reference] |  |
| Q2 | 1.18(1.07-1.30) | <0.001 | 1.40(1.25-1.57) | <0.001 | 1.15(1.00-1.33) | 0.055 |
| Q3 | 1.28(1.16-1.42) | <0.001 | 1.50(1.32-1.70) | <0.001 | 1.25(1.08-1.45) | 0.003 |
| Q4 | 1.42(1.28-1.58) | <0.001 | 1.54(1.34-1.77) | <0.001 | 1.37(1.17-1.59) | <0.001 |
| *P for trend* |  | <0.001 |  | <0.001 |  | <0.001 |

Abbreviation: OR, odds ratio; 95% CI, 95% confidence interval; NAFLD: nonalcoholic fatty liver disease.


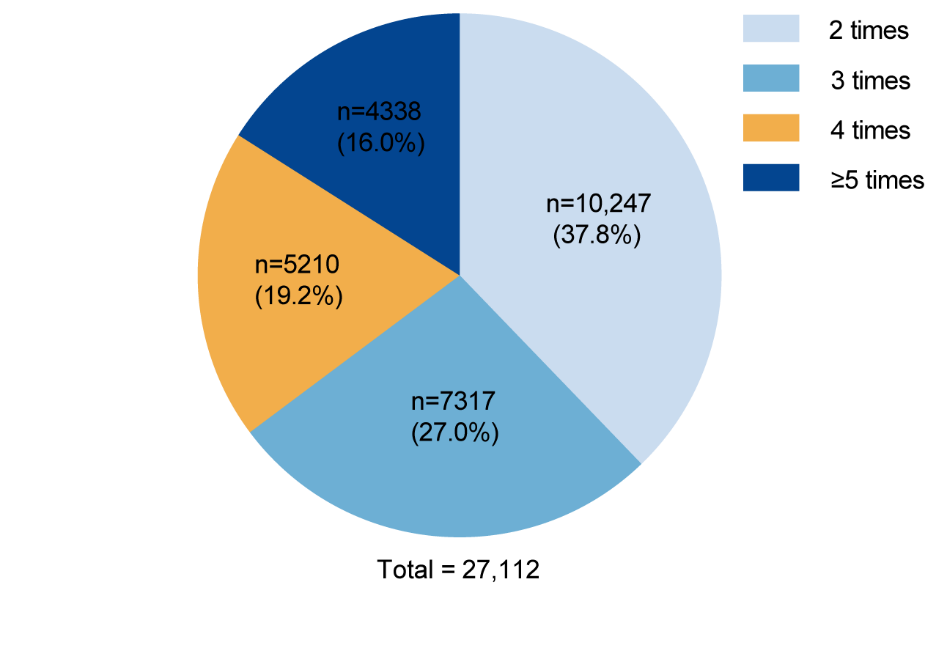


**Figure S1** Numbers of health check-up system visits of all eligible subjects.


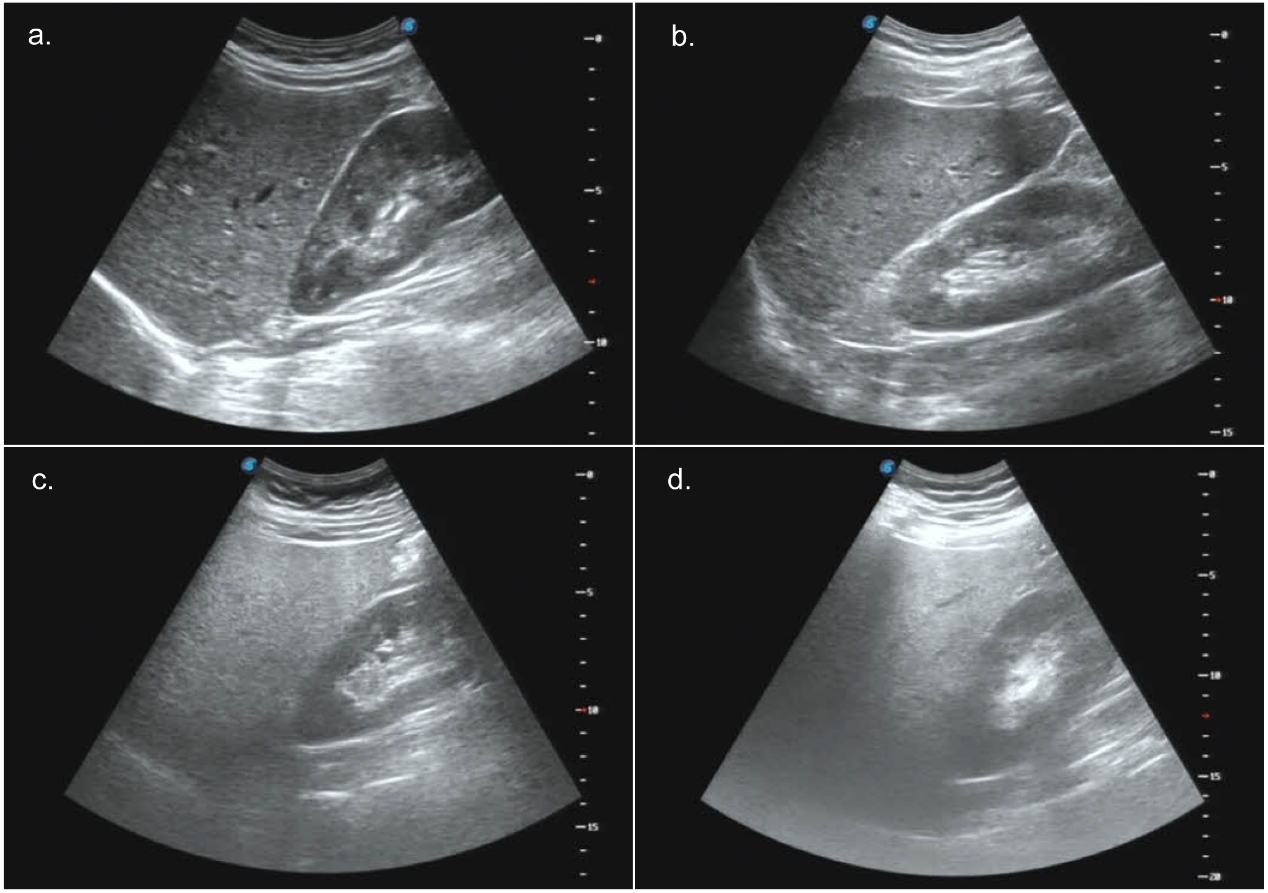


**Figure S2** Representative ultrasound images of non-, mild, moderate and severe NAFLD. a: Representative ultrasound images of non-NAFLD; b: Representative ultrasound images of mild NAFLD; c: Representative ultrasound images of moderate NAFLD; d: Representative ultrasound images of severe NAFLD.
